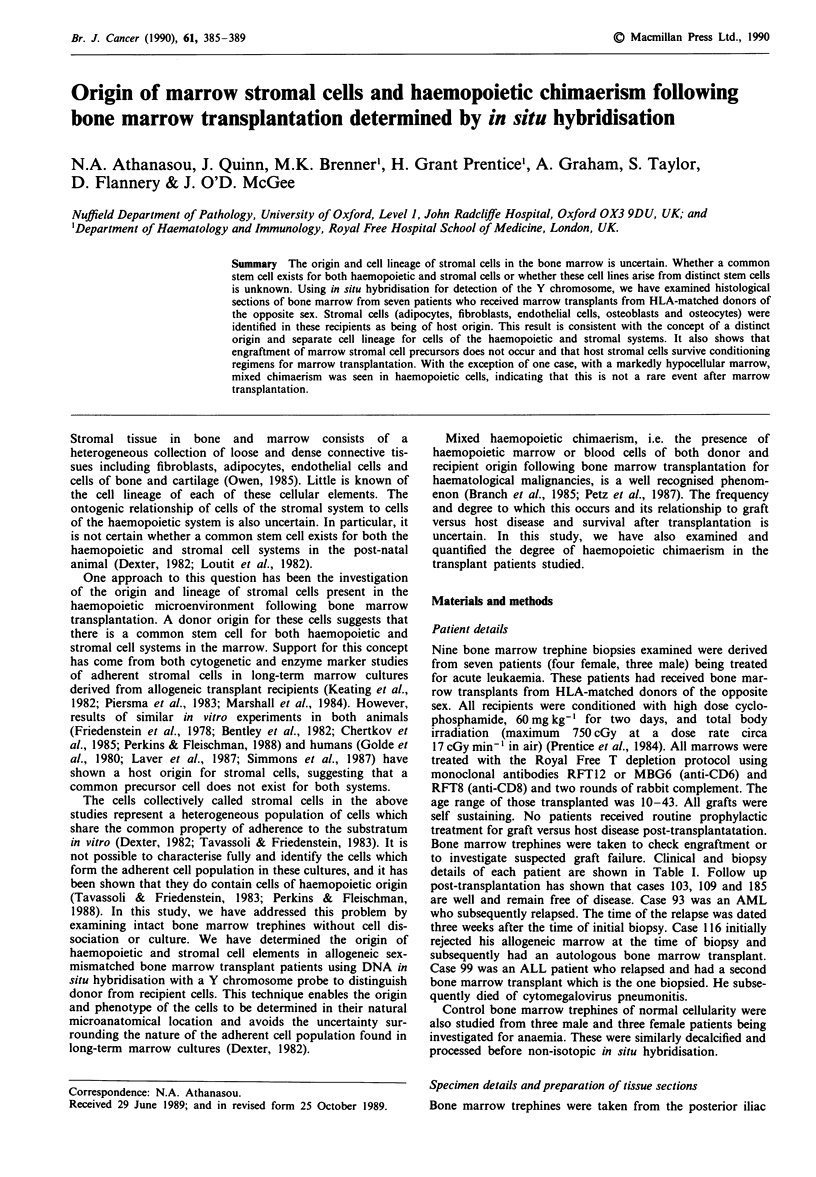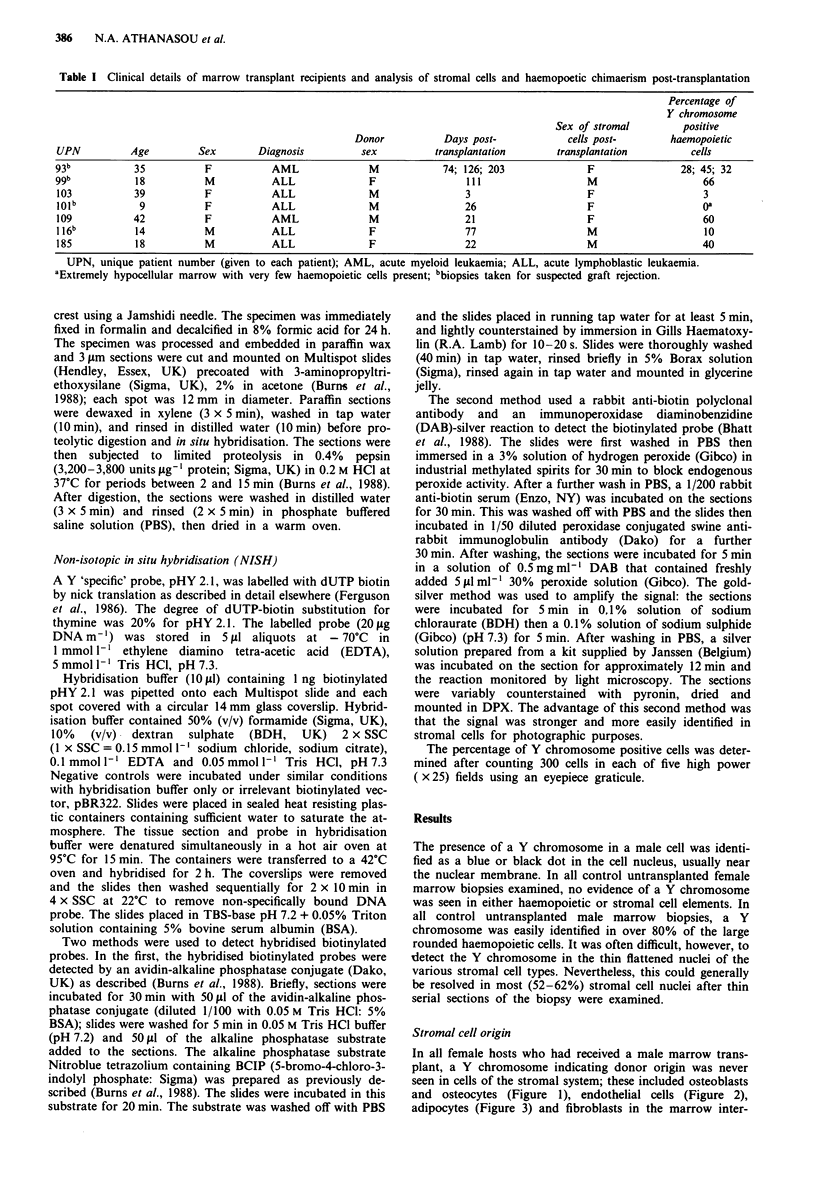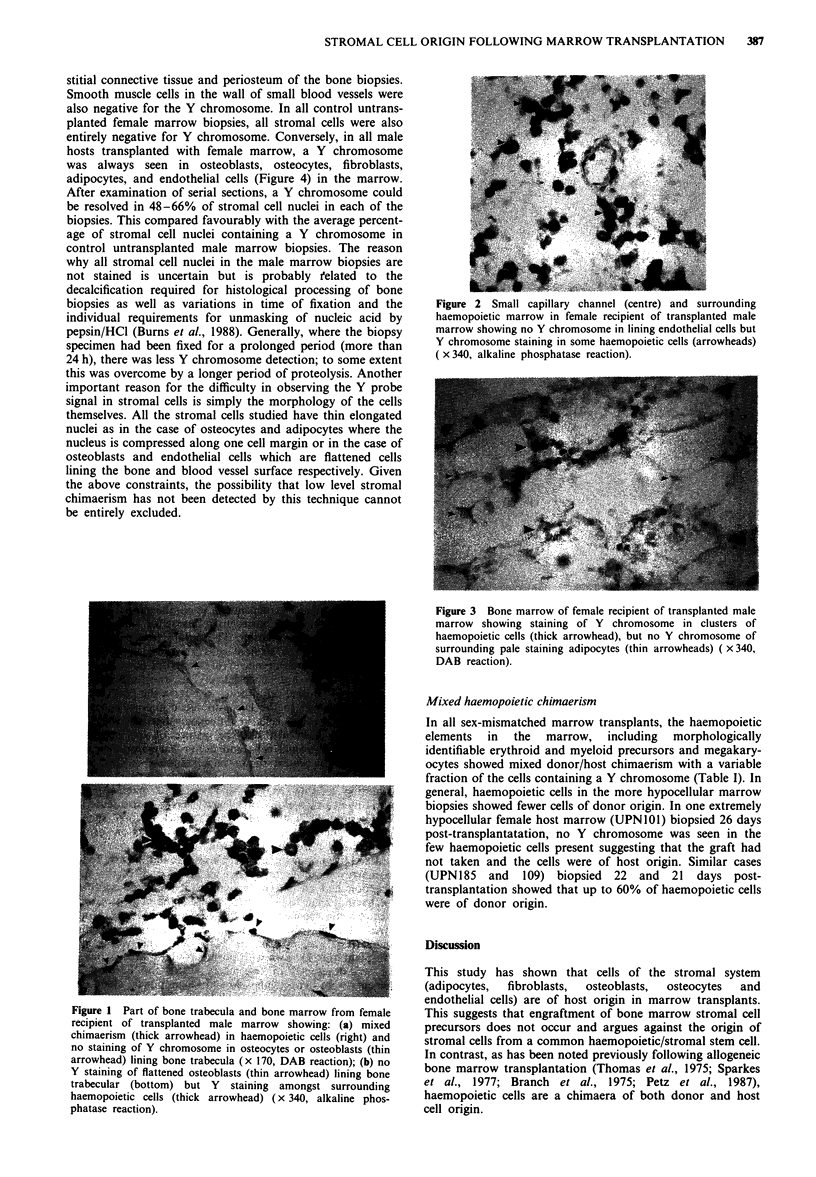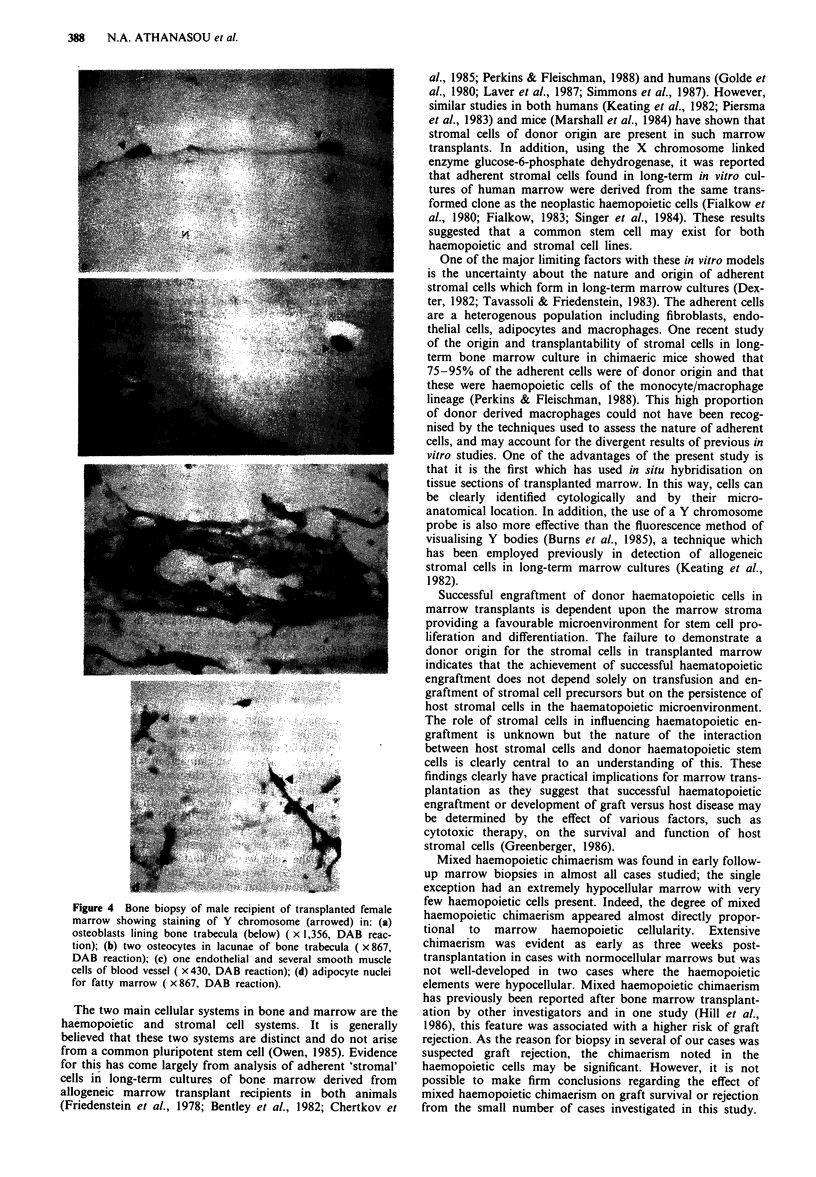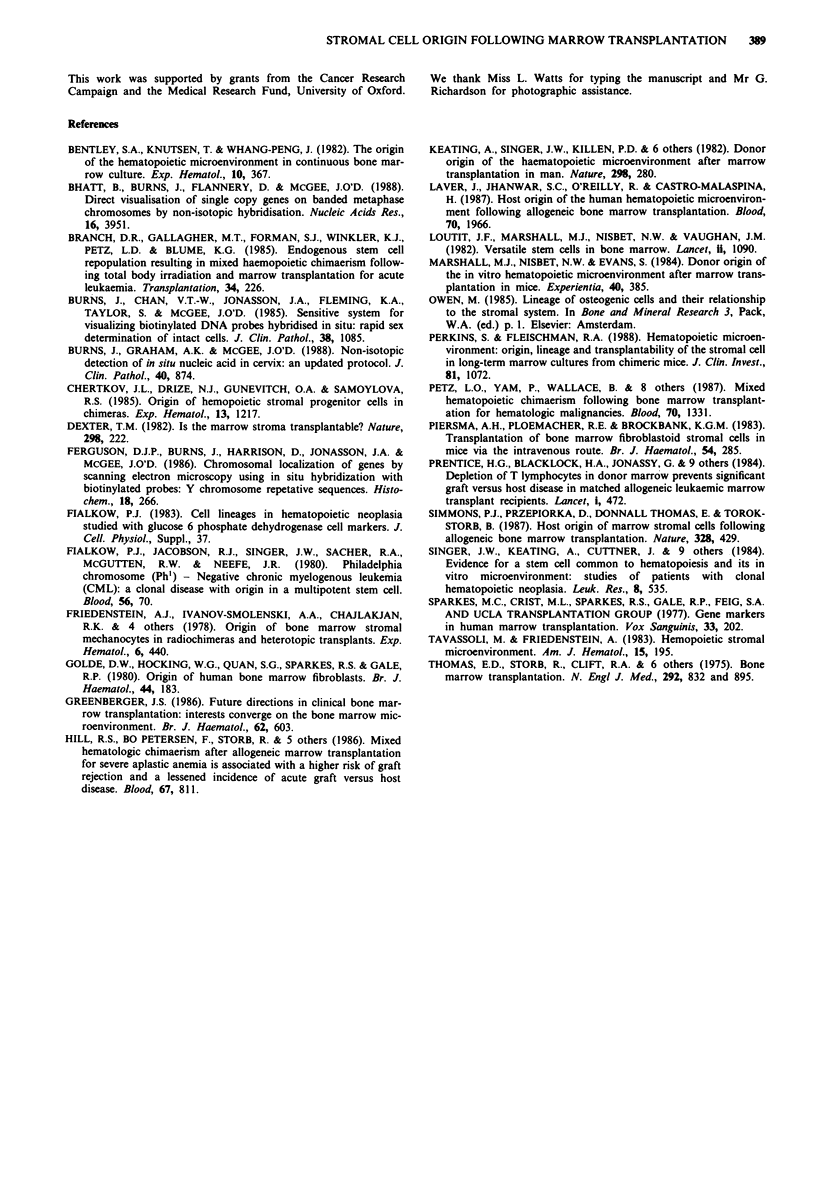# Origin of marrow stromal cells and haemopoietic chimaerism following bone marrow transplantation determined by in situ hybridisation.

**DOI:** 10.1038/bjc.1990.84

**Published:** 1990-03

**Authors:** N. A. Athanasou, J. Quinn, M. K. Brenner, H. G. Prentice, A. Graham, S. Taylor, D. Flannery, J. O. McGee

**Affiliations:** Nuffield Department of Pathology, University of Oxford, John Radcliffe Hospital, UK.

## Abstract

**Images:**


Br. J. Cancer (1990), 61, 385-389 © Macmillan Press Ltd., 1990

### Origin of marrow stromal cells and haemopoietic chimaerism following bone marrow transplantation determined by *in situ* hybridisation

N.A. Athanasou, J. Quinn, M.K. Brenner[1], H. Grant Prentice[1], A. Graham, S. Taylor, D. Flannery & J. O'D. McGee

*Nuffield Department of Pathology, University of Oxford, Level 1, John Radcliffe Hospital, Oxford OX3 9DU, UK; and [1]Department of Haematology and Immunology, Royal Free Hospital School of Medicine, London, UK.*

**Summary** The origin and cell lineage of stromal cells in the bone marrow is uncertain. Whether a common stem cell exists for both haemopoietic and stromal cells or whether these cell lines arise from distinct stem cells is unknown. Using *in situ* hybridisation for detection of the Y chromosome, we have examined histological sections of bone marrow from seven patients who received marrow transplants from HLA-matched donors of the opposite sex. Stromal cells (adipocytes, fibroblasts, endothelial cells, osteoblasts and osteocytes) were identified in these recipients as being of host origin. This result is consistent with the concept of a distinct origin and separate cell lineage for cells of the haemopoietic and stromal systems. It also shows that engraftment of marrow stromal cell precursors does not occur and that host stromal cells survive conditioning regimens for marrow transplantation. With the exception of one case, with a markedly hypocellular marrow, mixed chimaerism was seen in haemopoietic cells, indicating that this is not a rare event after marrow transplantation.

Stromal tissue in bone and marrow consists of a heterogeneous collection of loose and dense connective tissues including fibroblasts, adipocytes, endothelial cells and cells of bone and cartilage (Owen, 1985). Little is known of the cell lineage of each of these cellular elements. The ontogenic relationship of cells of the stromal system to cells of the haemopoietic system is also uncertain. In particular, it is not certain whether a common stem cell exists for both the haemopoietic and stromal cell systems in the post-natal animal (Dexter, 1982; Loutit *et al.*, 1982).

One approach to this question has been the investigation of the origin and lineage of stromal cells present in the haemopoietic microenvironment following bone marrow transplantation. A donor origin for these cells suggests that there is a common stem cell for both haemopoietic and stromal cell systems in the marrow. Support for this concept has come from both cytogenetic and enzyme marker studies of adherent stromal cells in long-term marrow cultures derived from allogeneic transplant recipients (Keating *et al.*, 1982; Piersma *et al.*, 1983; Marshall *et al.*, 1984). However, results of similar *in vitro* experiments in both animals (Friedenstein *et al.*, 1978; Bentley *et al.*, 1982; Chertkov *et al.*, 1985; Perkins & Fleischman, 1988) and humans (Golde *et al.*, 1980; Laver *et al.*, 1987; Simmons *et al.*, 1987) have shown a host origin for stromal cells, suggesting that a common precursor cell does not exist for both systems.

The cells collectively called stromal cells in the above studies represent a heterogeneous population of cells which share the common property of adherence to the substratum *in vitro* (Dexter, 1982; Tavassoli & Friedenstein, 1983). It is not possible to characterise fully and identify the cells which form the adherent cell population in these cultures, and it has been shown that they do contain cells of haemopoietic origin (Tavassoli & Friedenstein, 1983; Perkins & Fleischman, 1988). In this study, we have addressed this problem by examining intact bone marrow trephines without cell dissociation or culture. We have determined the origin of haemopoietic and stromal cell elements in allogeneic sex-mismatched bone marrow transplant patients using DNA *in situ* hybridisation with a Y chromosome probe to distinguish donor from recipient cells. This technique enables the origin and phenotype of the cells to be determined in their natural microanatomical location and avoids the uncertainty surrounding the nature of the adherent cell population found in long-term marrow cultures (Dexter, 1982).

Correspondence: N.A. Athanasou.
Received 29 June 1989; and in revised form 25 October 1989.

Mixed haemopoietic chimaerism, i.e. the presence of haemopoietic marrow or blood cells of both donor and recipient origin following bone marrow transplantation for haematological malignancies, is a well recognised phenomenon (Branch *et al.*, 1985; Petz *et al.*, 1987). The frequency and degree to which this occurs and its relationship to graft versus host disease and survival after transplantation is uncertain. In this study, we have also examined and quantified the degree of haemopoietic chimaerism in the transplant patients studied.

### Materials and methods

### *Patient details*

Nine bone marrow trephine biopsies examined were derived from seven patients (four female, three male) being treated for acute leukaemia. These patients had received bone marrow transplants from HLA-matched donors of the opposite sex. All recipients were conditioned with high dose cyclophosphamide, 60 mg $kg^{-1}$ for two days, and total body irradiation (maximum 750 cGy at a dose rate circa 17 cGy $min^{-1}$ in air) (Prentice *et al.*, 1984). All marrows were treated with the Royal Free T depletion protocol using monoclonal antibodies RFT12 or MBG6 (anti-CD6) and RFT8 (anti-CD8) and two rounds of rabbit complement. The age range of those transplanted was 10-43. All grafts were self sustaining. No patients received routine prophylactic treatment for graft versus host disease post-transplantatation. Bone marrow trephines were taken to check engraftment or to investigate suspected graft failure. Clinical and biopsy details of each patient are shown in Table I. Follow up post-transplantation has shown that cases 103, 109 and 185 are well and remain free of disease. Case 93 was an AML who subsequently relapsed. The time of the relapse was dated three weeks after the time of initial biopsy. Case 116 initially rejected his allogeneic marrow at the time of biopsy and subsequently had an autologous bone marrow transplant. Case 99 was an ALL patient who relapsed and had a second bone marrow transplant which is the one biopsied. He subsequently died of cytomegalovirus pneumonitis.

Control bone marrow trephines of normal cellularity were also studied from three male and three female patients being investigated for anaemia. These were similarly decalcified and processed before non-isotopic *in situ* hybridisation.

### *Specimen details and preparation of tissue sections*

Bone marrow trephines were taken from the posterior iliac

**Table I** Clinical details of marrow transplant recipients and analysis of stromal cells and haemopoetic chimaerism post-transplantation

| *UPN* | *Age* | *Sex* | *Diagnosis* | *Donor sex* | *Days post-transplantation* | *Sex of stromal cells post-transplantation* | *Percentage of Y chromosome positive haemopoietic cells* |
|---|---|---|---|---|---|---|---|
| 93[b] | 35 | F | AML | M | 74; 126; 203 | F | 28; 45; 32 |
| 99[b] | 18 | M | ALL | F | 111 | M | 66 |
| 103 | 39 | F | ALL | M | 3 | F | 3 |
| 101[b] | 9 | F | ALL | M | 26 | F | 0[a] |
| 109 | 42 | F | AML | M | 21 | F | 60 |
| 116[b] | 14 | M | ALL | F | 77 | M | 10 |
| 185 | 18 | M | ALL | F | 22 | M | 40 |

UPN, unique patient number (given to each patient); AML, acute myeloid leukaemia; ALL, acute lymphoblastic leukaemia. [a]Extremely hypocellular marrow with very few haemopoietic cells present; [b]biopsies taken for suspected graft rejection.

crest using a Jamshidi needle. The specimen was immediately fixed in formalin and decalcified in 8% formic acid for 24 h. The specimen was processed and embedded in paraffin wax and 3 μm sections were cut and mounted on Multispot slides (Hendley, Essex, UK) precoated with 3-aminopropyltriethoxysilane (Sigma, UK), 2% in acetone (Burns *et al.*, 1988); each spot was 12 mm in diameter. Paraffin sections were dewaxed in xylene (3 × 5 min), washed in tap water (10 min), and rinsed in distilled water (10 min) before proteolytic digestion and *in situ* hybridisation. The sections were then subjected to limited proteolysis in 0.4% pepsin (3,200–3,800 units $\mu g^{-1}$ protein; Sigma, UK) in 0.2 M HCl at 37°C for periods between 2 and 15 min (Burns *et al.*, 1988). After digestion, the sections were washed in distilled water (3 × 5 min) and rinsed (2 × 5 min) in phosphate buffered saline solution (PBS), then dried in a warm oven.

*Non-isotopic in situ hybridisation (NISH)*

A Y 'specific' probe, pHY 2.1, was labelled with dUTP biotin by nick translation as described in detail elsewhere (Ferguson *et al.*, 1986). The degree of dUTP-biotin substitution for thymine was 20% for pHY 2.1. The labelled probe (20 μg DNA $m^{-1}$) was stored in 5 μl aliquots at −70°C in 1 mmol $l^{-1}$ ethylene diamino tetra-acetic acid (EDTA), 5 mmol $l^{-1}$ Tris HCl, pH 7.3.

Hybridisation buffer (10 μl) containing 1 ng biotinylated pHY 2.1 was pipetted onto each Multispot slide and each spot covered with a circular 14 mm glass coverslip. Hybridisation buffer contained 50% (v/v) formamide (Sigma, UK), 10% (v/v) dextran sulphate (BDH, UK) 2 × SSC (1 × SSC = 0.15 mmol $l^{-1}$ sodium chloride, sodium citrate), 0.1 mmol $l^{-1}$ EDTA and 0.05 mmol $l^{-1}$ Tris HCl, pH 7.3 Negative controls were incubated under similar conditions with hybridisation buffer only or irrelevant biotinylated vector, pBR322. Slides were placed in sealed heat resisting plastic containers containing sufficient water to saturate the atmosphere. The tissue section and probe in hybridisation buffer were denatured simultaneously in a hot air oven at 95°C for 15 min. The containers were transferred to a 42°C oven and hybridised for 2 h. The coverslips were removed and the slides then washed sequentially for 2 × 10 min in 4 × SSC at 22°C to remove non-specifically bound DNA probe. The slides placed in TBS-base pH 7.2 + 0.05% Triton solution containing 5% bovine serum albumin (BSA).

Two methods were used to detect hybridised biotinylated probes. In the first, the hybridised biotinylated probes were detected by an avidin-alkaline phosphatase conjugate (Dako, UK) as described (Burns *et al.*, 1988). Briefly, sections were incubated for 30 min with 50 μl of the avidin-alkaline phosphatase conjugate (diluted 1/100 with 0.05 M Tris HCl: 5% BSA); slides were washed for 5 min in 0.05 M Tris HCl buffer (pH 7.2) and 50 μl of the alkaline phosphatase substrate added to the sections. The alkaline phosphatase substrate Nitroblue tetrazolium containing BCIP (5-bromo-4-chloro-3-indolyl phosphate: Sigma) was prepared as previously described (Burns *et al.*, 1988). The slides were incubated in this substrate for 20 min. The substrate was washed off with PBS and the slides placed in running tap water for at least 5 min, and lightly counterstained by immersion in Gills Haematoxylin (R.A. Lamb) for 10–20 s. Slides were thoroughly washed (40 min) in tap water, rinsed briefly in 5% Borax solution (Sigma), rinsed again in tap water and mounted in glycerine jelly.

The second method used a rabbit anti-biotin polyclonal antibody and an immunoperoxidase diaminobenzidine (DAB)-silver reaction to detect the biotinylated probe (Bhatt *et al.*, 1988). The slides were first washed in PBS then immersed in a 3% solution of hydrogen peroxide (Gibco) in industrial methylated spirits for 30 min to block endogenous peroxide activity. After a further wash in PBS, a 1/200 rabbit anti-biotin serum (Enzo, NY) was incubated on the sections for 30 min. This was washed off with PBS and the slides then incubated in 1/50 diluted peroxidase conjugated swine anti-rabbit immunoglobulin antibody (Dako) for a further 30 min. After washing, the sections were incubated for 5 min in a solution of 0.5 mg $ml^{-1}$ DAB that contained freshly added 5 μl $ml^{-1}$ 30% peroxide solution (Gibco). The gold-silver method was used to amplify the signal: the sections were incubated for 5 min in 0.1% solution of sodium chloraurate (BDH) then a 0.1% solution of sodium sulphide (Gibco) (pH 7.3) for 5 min. After washing in PBS, a silver solution prepared from a kit supplied by Janssen (Belgium) was incubated on the section for approximately 12 min and the reaction monitored by light microscopy. The sections were variably counterstained with pyronin, dried and mounted in DPX. The advantage of this second method was that the signal was stronger and more easily identified in stromal cells for photographic purposes.

The percentage of Y chromosome positive cells was determined after counting 300 cells in each of five high power (×25) fields using an eyepiece graticule.

### Results

The presence of a Y chromosome in a male cell was identified as a blue or black dot in the cell nucleus, usually near the nuclear membrane. In all control untransplanted female marrow biopsies examined, no evidence of a Y chromosome was seen in either haemopoietic or stromal cell elements. In all control untransplanted male marrow biopsies, a Y chromosome was easily identified in over 80% of the large rounded haemopoietic cells. It was often difficult, however, to detect the Y chromosome in the thin flattened nuclei of the various stromal cell types. Nevertheless, this could generally be resolved in most (52–62%) stromal cell nuclei after thin serial sections of the biopsy were examined.

*Stromal cell origin*

In all female hosts who had received a male marrow transplant, a Y chromosome indicating donor origin was never seen in cells of the stromal system; these included osteoblasts and osteocytes (Figure 1), endothelial cells (Figure 2), adipocytes (Figure 3) and fibroblasts in the marrow interstitial connective tissue and periosteum of the bone biopsies. Smooth muscle cells in the wall of small blood vessels were also negative for the Y chromosome. In all control untransplanted female marrow biopsies, all stromal cells were also entirely negative for Y chromosome. Conversely, in all male hosts transplanted with female marrow, a Y chromosome was always seen in osteoblasts, osteocytes, fibroblasts, adipocytes, and endothelial cells (Figure 4) in the marrow. After examination of serial sections, a Y chromosome could be resolved in 48–66% of stromal cell nuclei in each of the biopsies. This compared favourably with the average percentage of stromal cell nuclei containing a Y chromosome in control untransplanted male marrow biopsies. The reason why all stromal cell nuclei in the male marrow biopsies are not stained is uncertain but is probably related to the decalcification required for histological processing of bone biopsies as well as variations in time of fixation and the individual requirements for unmasking of nucleic acid by pepsin/HCl (Burns *et al.*, 1988). Generally, where the biopsy specimen had been fixed for a prolonged period (more than 24 h), there was less Y chromosome detection; to some extent this was overcome by a longer period of proteolysis. Another important reason for the difficulty in observing the Y probe signal in stromal cells is simply the morphology of the cells themselves. All the stromal cells studied have thin elongated nuclei as in the case of osteocytes and adipocytes where the nucleus is compressed along one cell margin or in the case of osteoblasts and endothelial cells which are flattened cells lining the bone and blood vessel surface respectively. Given the above constraints, the possibility that low level stromal chimaerism has not been detected by this technique cannot be entirely excluded.

**Figure 1** Part of bone trabecula and bone marrow from female recipient of transplanted male marrow showing: (**a**) mixed chimaerism (thick arrowhead) in haemopoietic cells (right) and no staining of Y chromosome in osteocytes or osteoblasts (thin arrowhead) lining bone trabecula ( × 170, DAB reaction); (**b**) no Y staining of flattened osteoblasts (thin arrowhead) lining bone trabecular (bottom) but Y staining amongst surrounding haemopoietic cells (thick arrowhead) ( × 340, alkaline phosphatase reaction).

**Figure 2** Small capillary channel (centre) and surrounding haemopoietic marrow in female recipient of transplanted male marrow showing no Y chromosome in lining endothelial cells but Y chromosome staining in some haemopoietic cells (arrowheads) ( × 340, alkaline phosphatase reaction).

**Figure 3** Bone marrow of female recipient of transplanted male marrow showing staining of Y chromosome in clusters of haemopoietic cells (thick arrowhead), but no Y chromosome of surrounding pale staining adipocytes (thin arrowheads) ( × 340, DAB reaction).

### *Mixed haemopoietic chimaerism*

In all sex-mismatched marrow transplants, the haemopoietic elements in the marrow, including morphologically identifiable erythroid and myeloid precursors and megakaryocytes showed mixed donor/host chimaerism with a variable fraction of the cells containing a Y chromosome (Table I). In general, haemopoietic cells in the more hypocellular marrow biopsies showed fewer cells of donor origin. In one extremely hypocellular female host marrow (UPN101) biopsied 26 days post-transplantatation, no Y chromosome was seen in the few haemopoietic cells present suggesting that the graft had not taken and the cells were of host origin. Similar cases (UPN185 and 109) biopsied 22 and 21 days post-transplantation showed that up to 60% of haemopoietic cells were of donor origin.

### Discussion

This study has shown that cells of the stromal system (adipocytes, fibroblasts, osteoblasts, osteocytes and endothelial cells) are of host origin in marrow transplants. This suggests that engraftment of bone marrow stromal cell precursors does not occur and argues against the origin of stromal cells from a common haemopoietic/stromal stem cell. In contrast, as has been noted previously following allogeneic bone marrow transplantation (Thomas *et al.*, 1975; Sparkes *et al.*, 1977; Branch *et al.*, 1975; Petz *et al.*, 1987), haemopoietic cells are a chimaera of both donor and host cell origin.

**Figure 4** Bone biopsy of male recipient of transplanted female marrow showing staining of Y chromosome (arrowed) in: (**a**) osteoblasts lining bone trabecula (below) ( × 1,356, DAB reaction); (**b**) two osteocytes in lacunae of bone trabecula ( × 867, DAB reaction); (**c**) one endothelial and several smooth muscle cells of blood vessel ( × 430, DAB reaction); (**d**) adipocyte nuclei for fatty marrow ( × 867, DAB reaction).

The two main cellular systems in bone and marrow are the haemopoietic and stromal cell systems. It is generally believed that these two systems are distinct and do not arise from a common pluripotent stem cell (Owen, 1985). Evidence for this has come largely from analysis of adherent 'stromal' cells in long-term cultures of bone marrow derived from allogeneic marrow transplant recipients in both animals (Friedenstein *et al.*, 1978; Bentley *et al.*, 1982; Chertkov *et al.*, 1985; Perkins & Fleischman, 1988) and humans (Golde *et al.*, 1980; Laver *et al.*, 1987; Simmons *et al.*, 1987). However, similar studies in both humans (Keating *et al.*, 1982; Piersma *et al.*, 1983) and mice (Marshall *et al.*, 1984) have shown that stromal cells of donor origin are present in such marrow transplants. In addition, using the X chromosome linked enzyme glucose-6-phosphate dehydrogenase, it was reported that adherent stromal cells found in long-term *in vitro* cultures of human marrow were derived from the same transformed clone as the neoplastic haemopoietic cells (Fialkow *et al.*, 1980; Fialkow, 1983; Singer *et al.*, 1984). These results suggested that a common stem cell may exist for both haemopoietic and stromal cell lines.

One of the major limiting factors with these *in vitro* models is the uncertainty about the nature and origin of adherent stromal cells which form in long-term marrow cultures (Dexter, 1982; Tavassoli & Friedenstein, 1983). The adherent cells are a heterogenous population including fibroblasts, endothelial cells, adipocytes and macrophages. One recent study of the origin and transplantability of stromal cells in long-term bone marrow culture in chimaeric mice showed that 75–95% of the adherent cells were of donor origin and that these were haemopoietic cells of the monocyte/macrophage lineage (Perkins & Fleischman, 1988). This high proportion of donor derived macrophages could not have been recognised by the techniques used to assess the nature of adherent cells, and may account for the divergent results of previous *in vitro* studies. One of the advantages of the present study is that it is the first which has used *in situ* hybridisation on tissue sections of transplanted marrow. In this way, cells can be clearly identified cytologically and by their microanatomical location. In addition, the use of a Y chromosome probe is also more effective than the fluorescence method of visualising Y bodies (Burns *et al.*, 1985), a technique which has been employed previously in detection of allogeneic stromal cells in long-term marrow cultures (Keating *et al.*, 1982).

Successful engraftment of donor haematopoietic cells in marrow transplants is dependent upon the marrow stroma providing a favourable microenvironment for stem cell proliferation and differentiation. The failure to demonstrate a donor origin for the stromal cells in transplanted marrow indicates that the achievement of successful haematopoietic engraftment does not depend solely on transfusion and engraftment of stromal cell precursors but on the persistence of host stromal cells in the haematopoietic microenvironment. The role of stromal cells in influencing haematopoietic engraftment is unknown but the nature of the interaction between host stromal cells and donor haematopoietic stem cells is clearly central to an understanding of this. These findings clearly have practical implications for marrow transplantation as they suggest that successful haematopoietic engraftment or development of graft versus host disease may be determined by the effect of various factors, such as cytotoxic therapy, on the survival and function of host stromal cells (Greenberger, 1986).

Mixed haemopoietic chimaerism was found in early follow-up marrow biopsies in almost all cases studied; the single exception had an extremely hypocellular marrow with very few haemopoietic cells present. Indeed, the degree of mixed haemopoietic chimaerism appeared almost directly proportional to marrow haemopoietic cellularity. Extensive chimaerism was evident as early as three weeks post-transplantation in cases with normocellular marrows but was not well-developed in two cases where the haemopoietic elements were hypocellular. Mixed haemopoietic chimaerism has previously been reported after bone marrow transplantation by other investigators and in one study (Hill *et al.*, 1986), this feature was associated with a higher risk of graft rejection. As the reason for biopsy in several of our cases was suspected graft rejection, the chimaerism noted in the haemopoietic cells may be significant. However, it is not possible to make firm conclusions regarding the effect of mixed haemopoietic chimaerism on graft survival or rejection from the small number of cases investigated in this study.

This work was supported by grants from the Cancer Research Campaign and the Medical Research Fund, University of Oxford. We thank Miss L. Watts for typing the manuscript and Mr G. Richardson for photographic assistance.

### References

BENTLEY, S.A., KNUTSEN, T. & WHANG-PENG, J. (1982). The origin of the hematopoietic microenvironment in continuous bone marrow culture. *Exp. Hematol.*, **10**, 367.

BHATT, B., BURNS, J., FLANNERY, D. & MCGEE, J.O'D. (1988). Direct visualisation of single copy genes on banded metaphase chromosomes by non-isotopic hybridisation. *Nucleic Acids Res.*, **16**, 3951.

BRANCH, D.R., GALLAGHER, M.T., FORMAN, S.J., WINKLER, K.J., PETZ, L.D. & BLUME, K.G. (1985). Endogenous stem cell repopulation resulting in mixed haemopoietic chimaerism following total body irradiation and marrow transplantation for acute leukaemia. *Transplantation*, **34**, 226.

BURNS, J., CHAN, V.T.-W., JONASSON, J.A., FLEMING, K.A., TAYLOR, S. & MCGEE, J.O'D. (1985). Sensitive system for visualizing biotinylated DNA probes hybridised in situ: rapid sex determination of intact cells. *J. Clin. Pathol.*, **38**, 1085.

BURNS, J., GRAHAM, A.K. & MCGEE, J.O'D. (1988). Non-isotopic detection of *in situ* nucleic acid in cervix: an updated protocol. *J. Clin. Pathol.*, **40**, 874.

CHERTKOV, J.L., DRIZE, N.J., GUNEVITCH, O.A. & SAMOYLOVA, R.S. (1985). Origin of hemopoietic stromal progenitor cells in chimeras. *Exp. Hematol.*, **13**, 1217.

DEXTER, T.M. (1982). Is the marrow stroma transplantable? *Nature*, **298**, 222.

FERGUSON, D.J.P., BURNS, J., HARRISON, D., JONASSON, J.A. & MCGEE, J.O'D. (1986). Chromosomal localization of genes by scanning electron microscopy using in situ hybridization with biotinylated probes: Y chromosome repetative sequences. *Histochem.*, **18**, 266.

FIALKOW, P.J. (1983). Cell lineages in hematopoietic neoplasia studied with glucose 6 phosphate dehydrogenase cell markers. *J. Cell. Physiol.*, Suppl., 37.

FIALKOW, P.J., JACOBSON, R.J., SINGER, J.W., SACHER, R.A., MCGUTTEN, R.W. & NEEFE, J.R. (1980). Philadelphia chromosome ($Ph^1$) – Negative chronic myelogenous leukemia (CML): a clonal disease with origin in a multipotent stem cell. *Blood*, **56**, 70.

FRIEDENSTEIN, A.J., IVANOV-SMOLENSKI, A.A., CHAJLAKJAN, R.K. & 4 others (1978). Origin of bone marrow stromal mechanocytes in radiochimeras and heterotopic transplants. *Exp. Hematol.*, **6**, 440.

GOLDE, D.W., HOCKING, W.G., QUAN, S.G., SPARKES, R.S. & GALE, R.P. (1980). Origin of human bone marrow fibroblasts. *Br. J. Haematol.*, **44**, 183.

GREENBERGER, J.S. (1986). Future directions in clinical bone marrow transplantation: interests converge on the bone marrow microenvironment. *Br. J. Haematol.*, **62**, 603.

HILL, R.S., BO PETERSEN, F., STORB, R. & 5 others (1986). Mixed hematologic chimaerism after allogeneic marrow transplantation for severe aplastic anemia is associated with a higher risk of graft rejection and a lessened incidence of acute graft versus host disease. *Blood*, **67**, 811.

KEATING, A., SINGER, J.W., KILLEN, P.D. & 6 others (1982). Donor origin of the haematopoietic microenvironment after marrow transplantation in man. *Nature*, **298**, 280.

LAVER, J., JHANWAR, S.C., O'REILLY, R. & CASTRO-MALASPINA, H. (1987). Host origin of the human hematopoietic microenvironment following allogeneic bone marrow transplantation. *Blood*, **70**, 1966.

LOUTIT, J.F., MARSHALL, M.J., NISBET, N.W. & VAUGHAN, J.M. (1982). Versatile stem cells in bone marrow. *Lancet*, **ii**, 1090.

MARSHALL, M.J., NISBET, N.W. & EVANS, S. (1984). Donor origin of the in vitro hematopoietic microenvironment after marrow transplantation in mice. *Experientia*, **40**, 385.

OWEN, M. (1985). Lineage of osteogenic cells and their relationship to the stromal system. In *Bone and Mineral Research 3*, Pack, W.A. (ed.) p. 1. Elsevier: Amsterdam.

PERKINS, S. & FLEISCHMAN, R.A. (1988). Hematopoietic microenvironment: origin, lineage and transplantability of the stromal cell in long-term marrow cultures from chimeric mice. *J. Clin. Invest.*, **81**, 1072.

PETZ, L.O., YAM, P., WALLACE, B. & 8 others (1987). Mixed hematopoietic chimaerism following bone marrow transplantation for hematologic malignancies. *Blood*, **70**, 1331.

PIERSMA, A.H., PLOEMACHER, R.E. & BROCKBANK, K.G.M. (1983). Transplantation of bone marrow fibroblastoid stromal cells in mice via the intravenous route. *Br. J. Haematol.*, **54**, 285.

PRENTICE, H.G., BLACKLOCK, H.A., JONASSY, G. & 9 others (1984). Depletion of T lymphocytes in donor marrow prevents significant graft versus host disease in matched allogeneic leukaemic marrow transplant recipients. *Lancet*, **i**, 472.

SIMMONS, P.J., PRZEPIORKA, D., DONNALL THOMAS, E. & TOROK-STORB, B. (1987). Host origin of marrow stromal cells following allogeneic bone marrow transplantation. *Nature*, **328**, 429.

SINGER, J.W., KEATING, A., CUTTNER, J. & 9 others (1984). Evidence for a stem cell common to hematopoiesis and its in vitro microenvironment: studies of patients with clonal hematopoietic neoplasia. *Leuk. Res.*, **8**, 535.

SPARKES, M.C., CRIST, M.L., SPARKES, R.S., GALE, R.P., FEIG, S.A. AND UCLA TRANSPLANTATION GROUP (1977). Gene markers in human marrow transplantation. *Vox Sanguinis*, **33**, 202.

TAVASSOLI, M. & FRIEDENSTEIN, A. (1983). Hemopoietic stromal microenvironment. *Am. J. Hematol.*, **15**, 195.

THOMAS, E.D., STORB, R., CLIFT, R.A. & 6 others (1975). Bone marrow transplantation. *N. Engl J. Med.*, **292**, 832 and 895.